# Unexpected intensive care transfer of admitted patients with severe sepsis

**DOI:** 10.1186/s40560-017-0239-7

**Published:** 2017-07-12

**Authors:** Gabriel Wardi, Arvin R. Wali, Julian Villar, Vaishal Tolia, Christian Tomaszewski, Christian Sloane, Peter Fedullo, Jeremy R. Beitler, Matthew Nolan, Daniel Lasoff, Rebecca E. Sell

**Affiliations:** 1 Department of Emergency Medicine and Division of Pulmonary, Critical Care and Sleep Medicine, UC San Diego Health System, 200 West Arbor Drive, San Diego, CA 92103 USA; 2 University of California San Diego School of Medicine, 9500 Gilman Drive, La Jolla, CA 92093-0602 USA; 3 Division of Pulmonary and Critical Care Medicine, Stanford University School of Medicine, M121-L, Stanford, CA 94305-5119 USA; 4 Department of Internal Medicine, University of California, San Diego, 200 West Arbor Drive, San Diego, CA 92103 USA; 5 Department of Emergency Medicine, University of California, San Diego, 200 West Arbor Drive, San Diego, CA 92103 USA; 6 Division of Pulmonary, Critical Care, and Sleep Medicine, University of California, San Diego, 200 W Arbor Drive, San Diego, CA 92103 USA

**Keywords:** Sepsis, Severe sepsis, Septic shock, Lactate, Unexpected ICU transfer, Mortality

## Abstract

**Background:**

Patients with severe sepsis generally respond well to initial therapy administered in the emergency department (ED), but a subset later decompensate and require unexpected transfer to the intensive care unit (ICU). This study aimed to identify clinical factors that can predict patients at increased risk for delayed transfer to the ICU and the association of delayed ICU transfer with mortality.

**Methods:**

This is a nested case-control study in a prospectively collected registry of patients with severe sepsis and septic shock at two EDs. Cases had severe sepsis and unexpected ICU transfer within 48 h of admission from the ED; controls had severe sepsis but remained in a non-ICU level of care. Univariate and multivariate regression analyses were used to identify predictors of unexpected transfer to the ICU, which was the primary outcome. Differences in mortality between these two groups as well as a cohort of patients directly admitted to the ICU were also calculated.

**Results:**

Of the 914 patients in our registry, 358 patients with severe sepsis were admitted from the ED to non-ICU level of care; 84 (23.5%) had unexpected ICU transfer within 48 h. Demographics and baseline co-morbidity burden were similar for patients requiring versus not requiring delayed ICU transfer. In unadjusted analysis, lactate ≥4 mmol/L and infection site were significantly associated with unexpected ICU upgrade. In forward selection multivariate logistic regression analysis, lactate ≥4 mmol/L (OR 2.0, 95% CI 1.03, 3.73; *p* = 0.041) and night (5 PM to 7 AM) admission (OR 1.9, 95% CI 1.07, 3.33; *p* = 0.029) were independent predictors of unexpected ICU transfer. Mortality of patients who were not upgraded to the ICU was 8.0%. Patients with unexpected ICU upgrade had similar mortality (25.0%) to those patients with severe sepsis/septic shock (24.6%) who were initially admitted to the ICU, despite less severe indices of illness at presentation.

**Conclusions:**

Serum lactate ≥4 mmol/L and nighttime admissions are associated with unexpected ICU transfer in patients with severe sepsis. Mortality among patients with delayed ICU upgrade was similar to that for patients initially admitted directly to the ICU.

## Background

Severe sepsis and septic shock are responsible for over 750,000 inpatient stays and over 215,000 deaths annually in the USA [1]. Early goal-directed therapy (EGDT) was shown to reduce mortality in patients with severe sepsis and septic shock compared to the then-standard therapy [2]. While further studies have challenged the necessity of strict adherence to the algorithms of EGDT as standard of care evolves, prompt identification, early administration of antibiotics, source control, and aggressive fluid resuscitation have improved outcomes for patients with sepsis [3–6]. Many emergency departments (ED) have adopted bundled care strategies to identify, treat, and improve management of sepsis, which have been shown to decrease mortality in these patients [7].

Some patients with severe sepsis respond well initially to aggressive care, but later decompensate. Early identification of this subset of patients could help ensure assignment to the appropriate level of care on admission and avoid subsequent delayed escalations of care associated with worse outcomes [8]. However, identification of these patients remains a challenge. Prior research has evaluated general patient characteristics for all-comers admitted from the ED at risk of delayed escalation to the ICU, and this includes tachypnea, sepsis, elevated lactate, non-sustained hypotension, and fever [9–12]. However, risk factors for unexpected decompensation and ICU transfer in patients admitted with severe sepsis have not been specifically examined.

The aim of the current study, therefore, is to identify risk factors that predict unexpected upgrade to the ICU within 48 h of hospital admission in patients with severe sepsis presenting to the ED and to quantify the association between delayed ICU upgrade and in-hospital mortality.

## Methods

### Study design, setting, definitions, and population

We performed a retrospective cohort study of patients presenting with severe sepsis or septic shock to two urban hospitals within the same university system, between July 2012 and September 2014. Our institutional review board approved this study with waiver of informed consent, IRB# 151413. One hospital is a quaternary care center while the other functions as a safety-net hospital with a total combined annual ED census of approximately 60,000 patients per year. Provider staffing of both emergency departments is based on expected patient census and did not change over the study period. Our hospital system adopted a bundled care initiative, referred to as “Code Sepsis,” designed to rapidly identify and treat patients with suspected severe sepsis or septic shock. The major components of our bundle care protocol are summarized in Table 1. Our institution had a 79% compliance with core measures of the protocol from 2012 to 2014, with an increase to 90% compliance by 2014.

**Table 1:** Summary of key aspects in our bundled care initiative

Identification:
A. Any 2 of the following (at least 2 required)
(1) Temp >38.3 °C (100.9 °F) or <36.0 °C (96.8 °F)
(2) Heart rate >90/min
(3) Respiratory rate >20 breaths per min
AND
B. Evidence of hypoperfusion (at least 1 required)
(1) MAP <65 mmHg
(2) SBP 40 mmHg below baseline
(3) Acutely altered mental status
(4) Oxygen saturation <92%
(5) Exam suggestive of hypoperfusion
AND
C. Suspected infection source
Management:
Phase 1:
Ensure adequate intravenous access
Weight-based IV fluid bolus
Repeat serum lactate 3 h after first specimen obtained
Administer broad-spectrum IV antibiotics in parallel
If persistent hypotension OR failure to clear lactate by 10%, start phase 2
Phase 2:
Obtain central venous access
Obtain ScvO2
Transduce CVC to measure a CVP
Insert arterial catheter
Additional volume resuscitation
Begin vasopressor
Contact nursing/house supervisor and ICU team
Serial lactate and Scv02 (every 6 h)
Consider transfusion to hematocrit of 30 if ScvO2 < 65% after volume resuscitation and pressor initiation
Consider corticosteroids if vasopressor-dependent hypotension

All patients identified by our bundled care initiative between July 2012 and September 2014 were eligible for inclusion. Patient data initially were reviewed and recorded by a senior critical care attending physician (P.F.) to confirm diagnosis of severe sepsis or septic shock. Patients were considered to have severe sepsis if they met all three of the following criteria: (1) at least two-fourths systemic inflammatory syndrome criteria (SIRS) (heart rate >90, white blood cell count >12 × 10^3^/μL or <4 × 10^3^/μL or >10% immature bands, temperature >38 °C or <36 °C, or respiratory rate >20), (2) either a confirmed or suspected infection, (3) and had evidence of end-organ damage defined as any one of the following: (a) bilateral pulmonary infiltrates with new (increased) oxygen requirement to keep saturation >90% or PaO_2_/FiO_2_ < 250 mm Hg in absence of pneumonia, <200 mm Hg in presence of pneumonia, (b) systolic blood pressure (SBP) <90 or mean arterial pressure <65 or SBP decrease >40 mm Hg from baseline, (c) urine output <0.5 mL/kg/h for >2 h or creatinine increase >2.0 mg/dL or doubling of baseline creatinine, (d) bilirubin >4.0 mg/dL, (e) platelets <80,000 × 10^3^/μL or >50% reduction from baseline, (f) international normalized ratio >1.5 or activated partial thromboplastin time >60 s, (g) pH < 7.30 or lactate > 4 mmol/L, or (h) acute alteration in mental status.

Septic shock was defined as a MAP < 65 mm Hg following a 20 mL/kg fluid bolus for at least 2 h or the need for vasoactive medications to ensure a MAP > 65 mm Hg.

The inclusion criteria were age ≥ 18, initiation of our bundled care plan during their ED stay, and admission to the wards from the ED. The exclusion criteria were admission directly to the ICU from the ED, direct admission/transfer to the wards without any care in the ED, initiation of our bundled care initiative for sepsis after admission to an inpatient unit, and patients with active hospice, comfort-only, or end-of-life care at the time of admission to the hospital. Our institution uses Society of Critical Care Medicine guidelines for admission to the ICU [13]. Thus, for patients with sepsis, ICU admission indications included the presence of hemodynamic instability and/or shock, the requirement for vasoactive medications or invasive blood pressure measurement, the need for mechanical ventilation, profound mental status changes, and/or a high level of nursing requirements only available in the ICU. Attending physician clinical judgment may override general guidelines dictating admission to the ICU.

Patients were classified as either “needing early escalation” or “not needing early escalation” based on their course over the first 48 h of admission. A 48-h window was selected based on prior studies have found that significant progression and potential decompensation of septic patients in the ED typically occurs by this point [11, 12]. Patients were classified as “needing early escalation” if admitted to a non-ICU level of care from the ED but subsequently upgraded to ICU level within the first 48 h or if they died in the wards within 48 h of admission. Patients were classified as “not needing early escalation” if no ICU upgrade was required during this time. Patients who required ICU care more than 48 h after admission to the wards were classified as “not needing early escalation.”

We evaluated the following candidate predictor variables for their association with needing early escalation: age, sex, initial and worrisome vital signs while in the ED (maximal heart rate, maximal temperature, maximal respiratory rate, minimal systolic blood pressure), maximal shock index (heart rate divided by systolic blood pressure), initial laboratory results in the ED (white blood cell count, serum bicarbonate, serum lactate, both as a continuous and dichotomized variable with a cutoff of 4 mmol/L, sodium, creatinine), Charlson co-morbidity index, length of stay in the ED (defined as from entry to the transition of care to the accepting service), day or night admission time (5 PM to 7 AM was considered a night admission), weekday (Monday to Friday) or weekend admission, residence in a nursing facility, active malignancy, immunosuppression (recent or chronic steroid use, HIV positive with CD4 < 200, organ transplantation, or active use of immunosuppressive medications), time to antibiotics after arrival to the ED, and volume of fluid administered per kilogram within 6 h of meeting criteria for severe sepsis or septic shock. Vital status at hospital discharge was also recorded. All data were reviewed and retrospectively extracted by three reviewers (G.W., A.W., and V.T.), who were involved in the study design and not blind to the study hypotheses. Data were abstracted into a standardized collection sheet devised by the authors (G.W., R.S.). Ten percent of charts were independently evaluated to assess inter-rater agreement and yielded a kappa greater than 0.85 for all variables.

### Statistical analysis

Data analysis was performed using SPSS Statistics version 22 (SPSS, Armonk, NY). Univariate analysis was performed using two-sample *t* tests, chi-squared tests and Fisher exact test as appropriate. A two-sided alpha of <0.05 was considered statistically significant. A forward selection multivariate logistic regression model was performed to identify independent predictors of ICU upgrade within 48 h. An entry probability of *F* set at 0.05 for entry and 0.10 for removal were used. For this model, the number of candidate predictor variables was limited to no more than 1 variable per 10 patients experiencing the primary endpoint. The following 8 variables were considered in the forward selection model-building process: serum lactate ≥4 mmol/L, presence of pneumonia, time to antibiotic administration, nighttime admission, Charlson co-morbidity index, shock index, quantity of fluid administered, and age. These variables were selected based upon prior studies that have shown that these are risk factors for unexpected ICU transfer, significant biological plausibility, or significant findings in the univariate analysis [10–12, 14]. A separate multivariable logistic model was used to determine if unexpected ICU transfer was an independent risk factor for death and included the following four variables: serum lactate ≥4 mmol/L, time to antibiotic administration, volume of fluids resuscitation, and shock index.

The sensitivity and specificity of select variables (initial serum lactate and night admission) found to be statistically significant in multivariate analysis were calculated. We also created a receiver operator characteristic curve to determine the optimal statistical cutoff point for initial serum lactate for maximal predictive value.

## Results

### Patient population characteristics

**Fig. 1:**
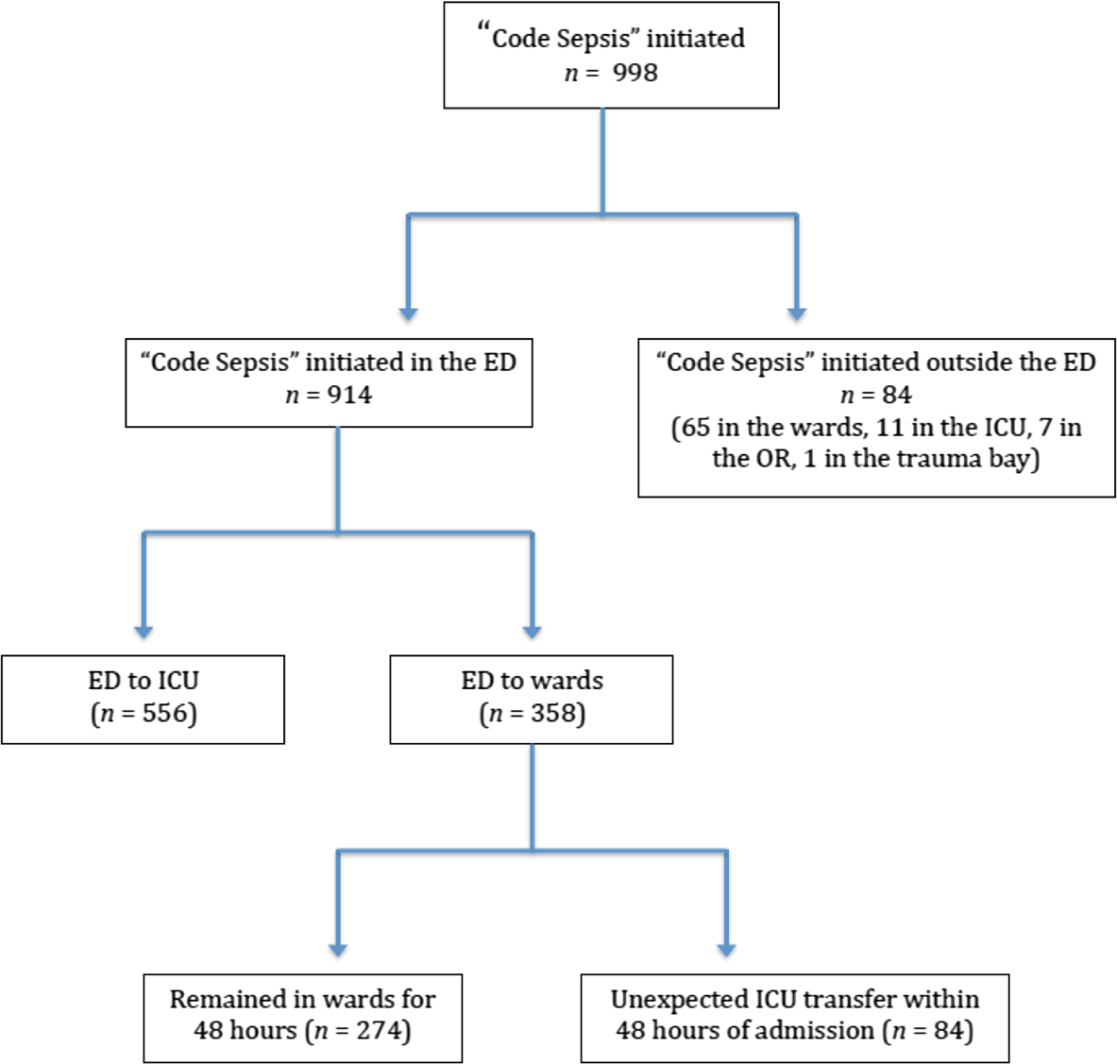
Breakdown of patients who received sepsis care in our bundled care initiative

Of the 998 patients who met the criteria for severe sepsis or septic shock, 914 were identified in the ED (Fig. 1). Of these, 358 had severe sepsis and were admitted to the wards. The remaining 556 patients were admitted directly to the ICU. When compared to patients directly admitted to the ICU from the ED, patients admitted to non-ICU level of care had lower mean initial lactate (2.8 vs. 4.1 mmol/L; *p* < 0.005) and shock index (1.02 vs. 1.11; *p* = .002) and received less fluid in the first 6 h (33 vs. 40 mL/kg; *p* < 0.005) as seen in Table 2.

**Table 2:** Baseline characteristics of patients with either severe sepsis or septic shock treated in the ED with our bundled care initiative

	ED directly to ICU	ED towards (including delayed ICU transfers within 48 h)	*p* value
Number	556	358	
Age, years	58.8 (±18)	58.4 (±18)	0.785
Sex, %male (*n*)	57.6% (320)	55.3% (198)	0.337
Triage SBP (mm Hg)	105 (±28)	112 (±28)	0.001
Shock index (SBP/HR)	1.11 (±0.33)	1.02 (±0.30)	0.002
Triage HR (BPM)	111 (±29)	110 (±23)	0.662
Fluids per kg within 6 h of presentation	40.1 (±20.7)	33.2 (±18.9)	<0.005
Initial lactate (mmol/L)	4.1 ± 3.4	2.8 ± 2.3	<0.005
% with septic shock at presentation (*n*)	55.6% (310)	0% (0)	<0.005
Site of infection%, (*n*)			<0.005
Abdominal	14.9% (83)	17.6% (63)	
Cardiac	1.3% (7)	1.1% (4)	
Central nervous system	1.1% (6)	1.4% (5)	
Genitourinary	21.9% (122)	31.3% (112)	
Musculoskeletal	7.0% (39)	13.4% (48)	
Pulmonary	24.8% (138)	22.3% (80)	
Unknown/others^a^	28.9% (161)	12.9% (46)	
Mortality,% (*n*)	24.6% (137)	12.0% (43)	<0.005

^a^Others include patients with multiple organ system infections, catheter-related infections, head and neck infection, and neutropenic fever without definitive source

### Predictors of early escalation to the ICU

Of the 358 patients admitted first to non-ICU level of care, 84 (23.5%) had an unexpected upgrade to the ICU within 48 h of admission. Univariate analysis (Table 3) revealed that higher initial lactate level was significantly associated with unexpected ICU transfer within 48 h (3.7 vs. 2.6 mmol/L; *p* = 0.011). The cutoff value of triage serum lactate levels that maximized correct prediction of unexpected ICU transfer was 4.44 mmol/L, which had a sensitivity of 0.282 and a specificity of 0.873. Serum lactate ≥4 mmol/L was found in 28.2% of patients with unexpected transfer versus in 16.7% of patients who remained in the wards (*p* = 0.039). The odds of experiencing early ICU transfer doubled for patients with an initial lactate level ≥4 mmol/L (OR 2.0, 95% CI 1.05, 3.66; *p* = 0.024). Patients with a genitourinary source were less likely to have delayed admission to the ICU; patients with pneumonia were more likely to decompensate on the floor (*p* = 0.03). Importantly, markers of bundle compliance and adherence to current resuscitation guidelines did not differ significantly as there was no difference in the time to antibiotic administration (96.6 vs. 113.1 min; *p* = 0.136) or volume of fluid administered in the first 6 h (35.7 vs. 32.4 mL/kg; *p* = 0.167) between the bundle compliant and bundle non-compliant groups.

**Table 3:** Results of univariate analysis of factors of unexpected ICU transfer in patients with severe sepsis initially admitted to the wards

	Early escalation to ICU (*n* = 84)	No escalation to ICU with 48 h (*n* = 274)	*p*
Patient characteristics			
Age, years	58.5 (±18.3)	58.2 (±16.0)	0.872
Sex, % male (*n*)	54% (45)	56% (153)	0.781
Charlson co-morbidity index	3.44 (±2.11)	3.01 (±2.51)	0.125
% immunocompromised (*n*)	67% (183)	58% (49)	0.152
% from nursing home (*n*)	13% (36)	14% (12)	0.855
% with active malignancy	30% (82)	32% (27)	0.787
Infection site,% (*n*)			0.03
Abdominal	17% (14)	18% (49)	
Cardiac	1% (1)	1% (3)	
Central nervous system	0% (0)	2% (5)	
Musculoskeletal	10% (8)	14% (39)	
Genitourinary	21% (18)	34% (94)	
Pulmonary	28% (24)	21% (56)	
Other/unknown/multiple	23% (19)	10% (27)	
Vital signs			
Triage temperature (°C)	37.3 (±1.0)	38.1 (±5.5)	0.190
Triage heart rate (BPM)	109 (±23)	110 (±24)	0.580
Triage systolic blood pressure (mm Hg)	113 (±27)	111 (±24)	0.612
Triage shock index (HR/SBP)	0.98 (±0.32)	1.03 (±0.28)	0.235
Minimum SBP during ED stay (mm Hg)	93 (±21)	94 (±21)	0.809
Maximal HR during ED stay (BPM)	120 (±24)	115 (±23)	0.072
Maximal RR during stay (RPM)	25 (±6)	25 (±11)	0.975
Laboratory results			
Sodium (mEq/L)	134 (±5)	133 (±11)	0.600
Creatinine (mg/dL)	1.70 (±1.5)	2.04 (±1.6)	0.692
Bicarbonate (mEq/L)	22 (±4.7)	23 (±4.3)	0.218
Lactate (mmol/L)	3.7 (±3.2)	2.6 (±1.8)	0.011
% with lactate > = 4 mmol/L	28.1%	16.7%	0.039
White count (×10^9^/L)	13.2 (±8.4)	11.3 (7.8)	0.051
Interventions			
Time to antibiotics (min)	96.6 (±89.9)	113.1 (±86.7)	0.136
Fluids administered in 1st 6 h (mL/kg)	35.7 (±18.5)	32.4 (±19.0)	0.167
Temporal impact			
Time in ED (min)	543 (±310)	646 (±411)	0.339
% admission during weekday (*n*)	70% (192)	76% (64)	0.334
% admission at night (*n*)	60% (50)	49% (134)	0.060
Mortality % (*n*)	25% (21)	8% (22)	<0.005

The sensitivity and specificity of lactate ≥4 mmol/L for early escalation to the ICU were 0.282 and 0.833, and night admission had a sensitivity and specificity of 0.595 and 0.509, respectively.

In forward selection multivariate logistic regression analysis, lactate ≥4 mmol/L (OR 2.0, 95% CI 1.03, 4.37; *p* = 0.003) and night admission (OR 1.9, 95% CI 1.07, 3.33; *p* = 0.029) were independently associated with early escalation to ICU level of care within 48 h of admission (Table 4).

**Table 4:** Results of forward selection multivariate logistic regression analysis to determine patient characteristics of unexpected ICU transfer

Variable	OR (95% CI)	*p*
Lactate ≥4 (mmol/L)	2.0 (1.03, 3.73)	0.041
Nighttime admission (5 PM—7 AM)	1.9 (1.07, 3.33)	0.029

OR are presented as odds of early escalation of care to the ICU

### Early ICU transfer and mortality

Overall, 43 of the 358 patients who were admitted to the wards (12.0%) did not survive to discharge. Death before discharge was significantly more likely among patients admitted to the wards who required early ICU upgrade compared to those without early upgrade (25 vs. 8%; *p* < .005). In unadjusted logistic regression analysis, early escalation to the ICU was associated with significantly higher mortality compared to non-escalation (OR 3.8, 95% CI 1.92, 7.20; *p* < 0.005). When adjusting for lactate ≥4 mmol/L, age, time to antibiotics, and SI, the association of delayed escalation with mortality remained highly significant (OR 4.2, 95% CI 1.87, 9.241; *p* < 0.005).

The in-hospital mortality of patients who were admitted from the ED directly to the ICU was 24.6%; this was nearly identical to the patients who had unexpected transfer to the ICU (*p* = 0.943). Despite this similar mortality rate, patients who had delayed ICU admission received less fluid volume administered in the first 6 h (35.7 mL/kg versus 40.1 mL/kg; *p* = 0.020) and lower shock index (0.99 versus 1.09; *p* = 0.013) and none were in shock at time of admission (0 versus 55.6%; *p* = <0.005). Serum lactate values were not statistically different however (3.7 versus 4.1 mmol/L; *p* = 0.357) nor was the percentage of patients with lactate ≥4 mmol/L (28.2% versus 34.3%; *p* = 0.351). In multivariate logistic regression analysis controlling for these variables, delayed ICU admission, compared to direct ICU admission, was not independently associated with increased mortality (OR 1.40, 95% CI 0.73, 2.67; *p* = 0.309).

## Discussion

Bundled care initiatives have been effective in the early identification and management of patients with severe sepsis and septic shock, yet some patients still unexpectedly decompensate and require ICU transfer after initial admission to the ward [7]. We found that an initial serum lactate ≥4 mmol/L was associated with a more than doubling of adjusted odds for early ICU transfer. Patients admitted between 5 PM and 7 AM were also more likely to have unexpected ICU upgrade in the multivariate logistic regression analysis. Patients with a delayed ICU transfer had a significant increase in mortality with an adjusted odds ratio of 4.2 compared to those that did not need early escalation; however, mortality was nearly identical when compared to patients directly admitted to the ICU.

Although numerous studies have evaluated factors associated with unexpected ICU admission, ours is unique in that it evaluates patients with suspected severe sepsis or septic shock who receive early, evidence-based aggressive care. Two prior studies have attempted to identify characteristics of infected patients from the ED that could predict unexpected transfer to the ICU. One evaluated patients with suspected infection (defined as blood cultures drawn in the ED or within 3 h of admission) and found that respiratory compromise, congestive heart failure, peripheral vascular disease, systolic blood pressure <100 mmHg, tachycardia, or elevated creatinine levels all predicted unanticipated ICU transfer within 48 h [15]. Caterino et al. studied patients who were given a discharge diagnosis of “sepsis” and performed a multivariate regression analysis of 78 patients and found that a lower bicarbonate and lack of fever were associated with unexpected ICU transfer [16].

Two previously published studies evaluated the development of septic shock in septic patients admitted from the ED. Glickman et al. investigated the progression of disease in hemodynamically stable septic patients without organ dysfunction to septic shock [11]. They found that the majority of patients who progressed to septic shock did so within 48 h and had an increased 30-day mortality versus in the group who did not progress to septic shock. The authors identified older age, female sex, presence of fever, anemia, comorbid lung disease, and vascular access device infection as risk factors. Capp et al. performed a retrospective chart review study to evaluate factors associated of the development of septic shock in septic patients admitted from the ED within the first 48 h [12]. The authors found approximately 8% of patients with sepsis progressed to septic shock at 48 h. Identified factors included a lactate >4 mmol/L, female gender, non-persistent hypotension, a bandemia of at least 10%, and a history of coronary artery disease. While our results have some similarities to these studies, ours is unique in that patients with sepsis (but not severe sepsis) were excluded and all patients received upfront, aggressive care. To our knowledge, no prior studies thus far have evaluated predictors of delayed ICU admission in a relatively ill patient population who received bundle care. That lactate >4 mmol/L was associated with the development of septic shock in the study by Capp further strengthens its use in determining which patients are at increased risk of unexpected ICU transfer. Furthermore, in the sensitivity analysis we performed to identify the serum lactate value that best predicts unexpected transfer to the ICU, we found that a lactate of 4.44 mmol/L had the best discriminatory value. We do not advocate adoption of this specific threshold in clinical decision-making, recognizing that sensitivity may be prioritized over specificity when screening for potentially fatal critical illness. Yet, this finding further strengthens using lactate values as a measure of severity of illness in septic shock. Indeed, prior research has shown that patients with serum lactate ≥4 mmol/L without hypotension had similar mortality to those with “overt” shock (defined as persistent hypotension after a 20 mL/kg fluid bolus) [17].

We also found that admission at night was an independent risk factor for unexpected ICU transfer. Staffing levels are lower at night in our ED, wards, and ICU that may partially explain this. Furthermore, certain hospital services, such as ultrasound and other diagnostic modalities, are not readily available at night, which may also contribute. This could be similar to the “weekend” effect—the increase in mortality seen in patients over the weekend compared to weekdays—that is well described in patients with acute medical conditions [18]. This “weekend” effect was also seen in a recent national database study that showed septic patients were more likely to have early in-hospital mortality—but not overall mortality—if admitted during the weekend [19]. Although the “weekend” effect was not shown in our analysis, we believe the similarities between this and night admission (e.g., lower staffing levels, less specialists and diagnostic modalities immediately available) suggests that patients fare poorer when less resources are available.

It has been previously shown that patients who have an unanticipated delayed upgrade to the ICU have higher mortality rates compared to those admitted to the ICU directly from the ED. Parkhe et al. found that a delayed ICU admission (defined as more than 24 h after initial admission) had a significantly higher mortality rate (35%) at 30 days than patients directly admitted to the ICU (9.1%) [20]. Similar studies have shown comparable results that are independent of the severity of illness at the time of admission [21]. However, in our cohort, delayed ICU admission was not independently associated with increased mortality. Differences in illness severity, threshold for ICU admission, inclusion of patients with septic shock, and hospital-specific sepsis protocols with early, aggressive may account for this discrepancy in findings.

### Limitations

We acknowledge several limitations in our study. It is retrospective and dependent upon chart review for data collected from a single hospital system, and thus, findings should be considered associations rather than causal relationships. As the patients in this study were identified by the medical staff in our ED, we suspect there were patients who met the inclusion criteria and would have benefited from aggressive care but did not have the bundled care initiative started. Furthermore, patients who received management in our bundled care initiative, but who did not meet criteria for severe sepsis or septic shock, were also excluded. We also did not include certain variables, such as bilirubin, degree of bandemia, lactate trends over time, and coagulation profile in our study, which prior research has shown to correlate with adverse events and increased mortality in patients with significant abnormalities in these variables [22, 23]. Finally, our data predate the most recent definition of sepsis and septic shock in our investigation (“Sepsis 3”) [24]. We acknowledge that the change in definitions could change the outcomes in our study. However, a high number of hospital systems use the “Sepsis 2” definitions of severe sepsis and septic shock, thus making our results applicable to general practice standards. Furthermore, numerous professional societies have not yet officially adopted the definitions in Sepsis 3.

## Conclusions

Serum lactate ≥4 and night admission were independently associated with increased probability of unexpected upgrade to the ICU in patients with severe sepsis. Unexpected ICU transfer was associated with a significant increase in mortality when compared to those in this population who remained in the wards for at least 48 h. Mortality was the same as for patients who were directly admitted to the ICU despite controlling for indices of illness in the group who were initially admitted to the wards. Prospective studies are needed to validate these results and this hypothesis.

## Abbreviations

ED: Emergency department
EGDT: Early goal-directed therapy
ICU: Intensive care unit
MAP: Mean arterial pressure
OR: Odds ratio
